# How can developing countries harness biotechnology to improve health?

**DOI:** 10.1186/1471-2458-7-346

**Published:** 2007-12-03

**Authors:** Abdallah S Daar, Kathryn Berndtson, Deepa L Persad, Peter A Singer

**Affiliations:** 1 The Program on Life Sciences, Ethics and Policy of the McLaughlin-Rotman Centre for Global Health, University Health Network | McLaughlin Centre for Molecular Medicine, University of Toronto, Ontario, Canada; 2 Department of Public Health Sciences, University of Toronto, Ontario, Canada; 3 Distinguished Investigator of the Canadian Institutes of Health Research, Department of Medicine at the University of Toronto, Ontario, Canada

## Abstract

**Background:**

The benefits of genomics and biotechnology are concentrated primarily in the industrialized world, while their potential to combat neglected diseases in the developing world has been largely untapped. Without building developing world biotechnology capacity to address local health needs, this disparity will only intensify. To assess the potential of genomics to address health needs in the developing world, the McLaughlin-Rotman Centre for Global Health, along with local partners, organized five courses on Genomics and Public Health Policy in the developing world. The overall objective of the courses was to collectively explore how to best harness genomics to improve health in each region. This article presents and analyzes the recommendations from all five courses.

**Discussion:**

In this paper we analyze recommendations from 232 developing world experts from 58 countries who sought to answer how best to harness biotechnology to improve health in their regions. We divide their recommendations into four categories: science; finance; ethics, society and culture; and politics.

**Summary:**

The Courses' recommendations can be summarized across the four categories listed above:

**Science:**

- Collaborate through national, regional, and international networks

- Survey and build capacity based on proven models through education, training, and needs assessments

**Finance:**

- Develop regulatory and intellectual property frameworks for commercialization of biotechnology

- Enhance funding and affordability of biotechnology

- Improve the academic-industry interface and the role of small and medium enterprise

**Ethics, Society, Culture:**

- Develop public engagement strategies to inform and educate the public about developments in genomics and biotechnology

- Develop capacity to address ethical, social and cultural issues

- Improve accessibility and equity

**Politics:**

- Strengthen understanding, leadership and support at the political level for biotechnology

- Develop policies outlining national biotechnology strategy

These recommendations provide guidance for all those interested in supporting science, technology, and innovation to improve health in the developing world. Applying these recommendations broadly across sectors and regions will empower developing countries themselves to harness the benefits of biotechnology and genomics for billions who have long been excluded.

## Background

Genomics and biotechnology hold great potential to fight diseases that disproportionately affect the world's poorest people. However, the benefits of biotechnology, driven by market incentives of the industrialized world, have accrued primarily to rich countries, with billions in the developing world largely excluded from these advances. Developing nations are now taking steps to build long-term plans to benefit from biotechnology innovation [1]. In Africa, the African Union Commission President developed a High-Level Panel on Modern Biotechnology to "generate a critical mass of technological expertise in targeted areas that offer high growth potential" from biotechnology and "harness biotechnology in order to develop Africa's rich biodiversity...improv [e] agricultural productivity and [develop] pharmaceutical products [2]." In January 2007, the African Ministerial Council on Science and Technology received the Panel report and committed themselves to a "20 year African Biotechnology Strategy" to promote that vision. The Federation of Asian Biotech Associations offers another Southern-based example of "collaboration between industry and academia" that seeks to "boost investment in biotechnology, international trade in biotechnology products, and outsourcing of services [3]." The need for developing countries to develop and benefit from biotechnology is clear – as a discussion paper from the World Bank's recent Global Forum on Science, Technology, and Innovation (STI) states, there is no longer a question of whether countries should build science and technology capacity that promotes biotechnology innovation, "but what type of capacity to build, given their economic constraints, and how best to implement these capacity building action plans [4]."

Driven by a mission to harness the advances of innovative technology for global health equity, the McLaughlin-Rotman Centre for Global Health (MRC), formerly the Canadian Program on Genomics and Global Health, sought to ask how developing countries can best harness health biotechnology to improve health in their regions. We define'genomics' as the powerful new wave of health-related life sciences (biotechnologies) energized by the Human Genome Project and the knowledge and tools it is spawning (including proteomics, transcriptomics, metabolomics, etc). Our operational definition encompasses the ethical, legal, social and cultural dimensions of developing the science and technologies and taking them to where they were needed: from the lab to the village, as it were. In this paper we use the terms 'biotechnology' and 'genomics' interchangeably. We first explored ways to harness biotechnology to improve the health in the developing world in 2001, followed by a study that identified the top ten biotechnologies for improving health in developing countries in 2002 [5,6]. Between 2002 and 2004, the MRCGH planned, developed, and executed five Executive Courses on Genomics and Public Health Policy in five regions in the developing world. In this endeavor we collaborated with local experts and institutions to bring together 232 developing world experts and key stakeholders from multiple sectors to determine the best way to harness genomics and health biotechnology to improve the health of people in the developing world. Previous recommendations on how to bring the benefits of biotechnology to the poor have not focused on generating broadly applicable guidelines for improving health, but rather on enhancing particular technologies, such as agricultural biotech [7,8] and nanotechnology [9], or providing action steps for particular nations [10] or stakeholders (e.g. civil society or research institutes) involved in promoting biotechnology [11]. Moreover, rather than employing wide-spread consultation with developing world experts to generate recommendations, existing proposals have come from small-scale workshops without a developing world focus [12,13], forums emphasizing development as opposed to health, or publications by lone developing world voices [9]. To our knowledge, never before has such a large, multi-sectoral, Southern-based group of experts been consulted on these issues. This paper offers a cross-comparison of their recommendations. The similitude of these independently generated recommendations supports their robustness as answers to the five courses' overarching question: how can the developing world best harness genomics and biotechnology to improve health?

## Discussion

### Executive Courses on Genomics and Public Policy

The Executive Courses on Genomics and Public Health Policy took place between 2002 and 2004 for experts in five regions of the developing world: Nairobi, Kenya with the African Centre for Technology Studies for the African continent; Kerala, India with Indian Council of Medical Research for the Indian subcontinent; Muscat, Oman with the World Health Organization's Regional Office for the Eastern Mediterranean region (EMRO); Caracas, Venezuela with the United Nations University's Biotechnology for Latin America and the Caribbean (BIOLAC) and the Pan American Health Organization for Latin America and the Caribbean; and Hong Kong SAR China with the University of Hong Kong for the Western Pacific and Southeast Asia region.

**Figure 1:**
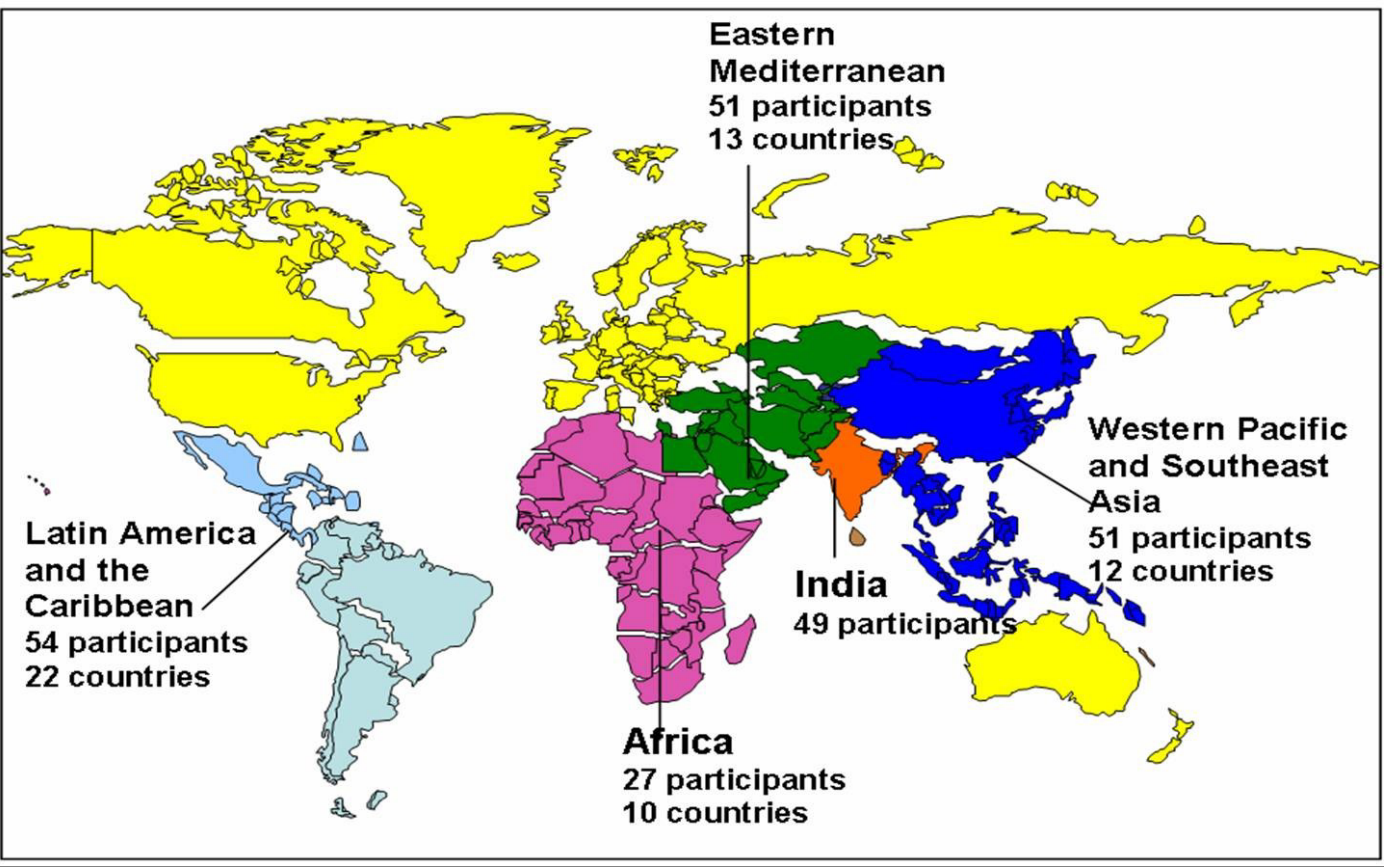
Regional breakdown of participants in the Executive Courses on Genomics and Public Health Policy.

The 232 participants from 58 countries (see Figure 1) were chosen based on contacts identified through our previous work related to this area including recommendations from field experts leading to a subsequent snowball effect. Thorough in-depth literature review and internet-based searches were also conducted to select and validate our participant choices. The participants were carefully selected to represent a wide range of interests relevant to biotechnology, with special consideration given to appropriately balancing geographical, gender and discipline/specialty distribution. The sectors represented included:

- government representatives, health ministry officials,

- regulatory officials, legal experts,

- scientists from academic institutions and industry, including the director of a national institute of genomics in the developing world, and a member of the research team that in 1997 cloned Dolly the sheep, the first animal ever cloned from an adult mammalian cell,

- industry executives, biotechnology company representatives,

- members of non-governmental organizations (NGOs), and media.

The executive courses had three goals:

1. To familiarize the participants with the current status and implications of health genomics and biotechnology, and to provide information relevant to public policy-making in these fields.

2. To provide frameworks for analyzing and debating the policy issues and related ethical questions in health genomics and biotechnology, and to help people to understand, anticipate and influence the legal and regulatory frameworks under which health biotechnology industries will operate, both nationally and internationally.

3. To begin developing a leaders' network reaching across different sectors (including industry, academic, government and NGOs) by sharing perspectives and building relationships.

The courses, each lasting four, intensive, interactive days, consisted of a series of presentations, primarily delivered by local experts, and discussions led by stakeholders from different countries and sectors, allowing for the opportunity to express different viewpoints They provided opportunities to share information about the research, ethics, social context, infrastructure, media relations, business development, and regulations affecting the development of biotechnology, and gave the participants background information. However, the main question, 'how best to harness biotechnology to improve global health,' and the regional recommendations were developed in small and large group discussions of the participants. Topics included scientific advances in biotechnology, innovations in business models, public sector perspectives, ethics, legal issues, and national innovation systems. This information is critical for developing countries if they are to absorb and control research information and public policy issues affecting major technological breakthroughs in the life sciences and public health.

Participants drove the process of identifying and collecting these recommendations. Participants constructed the recommendations by:

1. pre-drafting recommendations based on presentations;

2. deleting any recommendations which the group did not support;

3. adding missing recommendations;

4. sharpening recommendation language; and

5. attaining general or widespread agreement among participants.

Participants received evaluation forms at the end of each course. The general consensus reflected success in terms of achieving the goals and objectives of the Courses, with satisfaction ratings by the participants ranging from 86%-96%. The first three courses have been published individually without a synthesis of the recommendations as a whole [14-16]. This paper, however, compares and analyzes recommendations from all five courses.

### Recommendations from the Course Participants

In each course, working groups were asked for advice on developing genomics/biotechnology in the region to improve public health as outlined above. One of the products from each course was a set of recommendations on how best to harness biotechnology to address local health needs within their region. The recommendations are intended for use by policy-makers, industry leaders, scientists, health care providers, NGOs, and funding agencies. We applied these categories retrospectively to the Courses after developing them in consultation with developing world key informants in 2006. We compared and analyzed the Courses' recommendations and synthesized them into four categories as presented below: 1) science, 2) finance, 3) ethics, society, and culture, and 4) politics. These categories, while not completely mutually exclusive, help to present the recommendations of the 5 groups.

### Science

Within the recommendations related to science, participants focused on the potential of inter-sectoral, regional, and international collaboration to build capacity, the need for surveys of current capacity, and the importance of looking to successful models elsewhere. Participants specifically called for collaboration and capacity-building as methods to improve science education and establish regional and international networks – these networks possess the much needed capacity to increase dialogue between biotechnology developers and end-users. India's lack of emphasis on regional collaboration is likely linked to the fact that it was the only Course whose participants all came from one nation. Africa and Latin America, whose biotechnology capacities are comparatively less developed, both encouraged their regions to look to successful models of biotechnology innovation elsewhere ([17] see Table 1).

**Table 1:** Scientific Recommendations

**Africa**	**India**	**Eastern Mediterranean**	**Latin America and the Caribbean**	**Western Pacific/Southeast Asia**
-**Establish a regional network **to foster sustained **inter-sectoral dialogue** - Commission African **capacity survey in genomics-related R&D **to determine areas of strength - **Undertake a detailed study of R&D models **with demonstrated success in the developing world - Establish seven **regional research centres of excellence**	- Improve **industry-academic interface **with appropriate incentives to improve public health and the nation's wealth - Establish an internet-based opinion leaders' network to **foster cross-sectoral dialogue**	- Provide **coordination and networking among national biotechnology bodies **and coordinators to exchange information, expertise, and training - **Regional cooperation **in production and utilization of health biotechnology - Coordinate a **national survey/inventory/situation analysis/needs assessment of health biotechnology innovation systems**, including scientific and management capacity, government policies, legislation and regulations, intellectual property policies, private sector activity, and strengths/weaknesses, opportunities and threats - Encourage academic institutions to **include health biotechnology topics within **their **curricula **and **create specialized programs **and degrees where appropriate. There should be particular emphasis on ICT and bioinformatics	- Conduct a study both to **document **system's **strengths in genomics and biotechnology **and also to determine the needs which can be addressed by these disciplines - **Educate **and prepare the necessary **human resources **in genomics and biotechnology - **Seek help and advice from institutions in other countries **in the region that have had a successful experience in this endeavor -Develop **mechanisms of regional cooperation **to harness genomics and biotechnology for both health and economic development - **Harness the potential **of Latin America and the Caribbean in genomics and biotechnology to improve health for the population of the region - **Build on existing networks **so as to avoid duplications and redundancies - **Encourage **the **participation **of researchers, government officials, members of the private sector, members of civil society, and any other relevant stakeholders - **Address local health needs** - Should not only pursue pure research but also **applied problem solving investigation **and product development - **Facilitate learning** - A **strategy and a plan of action **should be built at the **regional level **in order to promote the creation of international, interdisciplinary, multidisciplinary, and multi-institutional projects	- Seek development funds from **national, regional and international sources** - **Perform foresight exercises**, including prioritization, needs assessment and action plan - **Facilitate linkages **between government, academia, NGO's, civil society, researchers, the health system and industry - **Build capacity **and share core facilities - Develop **joint training programs** - **Identify existing genomics/biotechnology capacity **including trained personnel, equipment, etc. in all public and private sectors - **Build essential core research facilities **linked to local needs - **Develop training programs **for different personnel categories - **Integrate genomics/biotechnology in curricula **beginning at a primary level to postgraduate levels

### Finance

Key issues that arose regarding finance included regulatory systems, intellectual property rights, and private sector collaboration. Participants stressed the need to harness the power of biotechnology not only for health, but also for economic development. Several regions stressed the need to identify appropriate entry points for biotechnology products and exploit domestic and regional markets [6]. India's lack of emphasis on product entry may be due to the fact that its private sector's affordable pharmaceuticals have already emerged competitively in domestic and global markets ([18] see Table 2).

**Table 2:** Financial Recommendations

**Africa**	**India**	**Eastern Mediterranean**	**Latin America and the Caribbean**	**Western Pacific/Southeast Asia**
- Create **sustainable financing mechanisms**	- Develop **independent, accountable, transparent regulatory systems **[...] for a single entry, smart and effective system - Improve **industry-academic interface **with appropriate incentives to improve public health and the nation's wealth - Increase **[government] funding **for healthcare research with appropriate emphasis on genomics	- Coordinate a national survey/inventory/situation analysis/needs assessment of health biotechnology innovation systems, **regulations, intellectual property policies, private sector activity, **and strengths/weaknesses, opportunities and threats - Develop a proposal for a **Regional Genomics and Health Research Fund **emphasizing both peer-reviewed research and capacity strengthening - The National Commission on Biotechnology, in collaboration with the relevant ministries, should **develop a plan to integrate genetic and genomics products **(including diagnostics, vaccines, therapies, and other genomic priorities), within the health system and public health programs	- Develop mechanisms of regional cooperation to harness genomics and biotechnology for both health and **economic development**. The networks should not only pursue pure research but also applied problem solving investigation and **product development** - Facilitate the development of guidelines on **intellectual property, **biosafety, bioethics, **regulation**, and public awareness - Adopt a **strategic point of entry **into genomics and biotechnology. Bioinformatics is one such potential point of entry; others should be identified through foresight exercises conducted in the region - **Domestic small and medium enterprises **in the region should form strategic alliances and **joint ventures**, with special emphasis on bringing together organizations from different countries in the region	- Identification of **cheaper alternative sources of energy** - Develop and harmonize **regulatory policies including IP**, biodiversity management, biosafety, movement of genetic material, protection of indigenous knowledge - Create **innovative business models **for economic and health care development and to support research - Identify **appropriate entry points **for genomics/biotechnology (e.g. various forms of agriculture, genetic screening, traditional medicine, bioinformatics, diagnostics for infectious diseases, etc) - Explore formation of **public-private partnerships **to address regional and national health needs

### Ethics, Society and Culture

Courses commenting on ESC issues called for public engagement programs that would inform and educate their populations on biotechnology developments. Another common theme included the need for capacity to address ethical issues including legal, social, and environmental concerns. Participants also underscored themes of accessibility and equity in terms of disseminating biotechnology innovations (see Table 3).

**Table 3:** ESC Recommendations

**Africa**	**India**	**Eastern Mediterranean**	**Latin America and the Caribbean**	**Western Pacific/Southeast Asia**
**N/A**	- **Engage the public **and ensure broad-based input into policy setting - **Ensure equitable access **of poor to genomics products and services - Develop independent, accountable, transparent regulatory systems... based on ethics to **ensure that ethical, legal and social issues are addressed**	- The National Commission on Biotechnology should **develop programs of public awareness and engagement**. Important "publics" here include media and religious leaders as well as the public at large. The discussion should **include ethical issues** - The emphasis should be on **accessibility and equity **to improve the health of the poor	- The **population **of the countries in the region **should be informed and educated **about the developments in genomics and biotechnology, and in the impact of these disciplines in addressing local health needs	- **Engage their publics **in ways that inform, seek feedback and use the feedback to inform policy - Increase capacity for research and development on **ethical, legal, environmental and social implications**

### Politics

Political recommendations from participants highlighted political leadership as a core factor in promoting biotechnology research and development in their regions. Many participants stressed the need for national strategy and public policy on genomics and biotechnology. African participants recommended using the established New Partnership for Africa's Development (NEPAD) as an entry point onto the continent's political agenda. Several regions also stressed the need for government support in funding and developing biotechnology (see Table 4).

**Table 4:** Political Recommendations

**Africa**	**India**	**Eastern Mediterranean**	**Latin America and the Caribbean**	**Western Pacific/Southeast Asia**
- Identify **champions among politicians** - Use the New Plan for African Development (NEPAD) as **entry point onto political agenda**	- Increase **[government] funding **for healthcare research with appropriate emphasis on genomics	- **Create National Commission **on Genomics, Biotechnology and Health, multisectoral membership include youth, women, and civil society. The focus should include ethical issues - Based on evidence from the national survey described above, governments of member states should develop and **adopt**, at the highest level, a **national biotechnology strategy** - Regional Director EMRO may be requested to **address the governments at the highest level **for actively considering the proposals of this workshop and for giving priority attention to genomics for health and health biotechnology. The **political leadership may be provided effective advocacy material**, with special reference to its link with poverty alleviation, public health objectives, and need for transfer (and internalization) of technology	- **Create**, at the local and regional levels, **a strategy to strengthen capacity **in science, in technology, and in management -**Establish a concrete genomics and biotechnology public policy**, a strategy, and a plan of action to develop and use these disciplines to address the country's most pressing health problems - **Build strategy at the regional level **in order to promote the creation of international, interdisciplinary, multidisciplinary, and multi-institutional projects	-Coordinate/undertake/conduct **strategic planning **aimed at achieving sustainability of programs using measurable benchmarks for desired regional and national outcomes - Encourage **proactive government support** - Advocate for **public **and private support

### Summary

Although the recommendations from the five courses display nuances linked to regional differences in biotechnology capacity and development, financial conditions, political frameworks, and population needs, fundamental lessons emerge from their insights. These lessons reinforce the results of another study rooted in developing world expert insights that highlighted the same four key forces: science; finance; ethics, society, and culture; and politics. The similitude of the Courses' recommendations, despite their independent generation in five different regions by over 200 participants, affirms the robustness of our answer to how genomics and biotechnology can best serve the health of the world's poorest people. Below is a summary of their recommendations based on those groupings (see Table 5).

**Table 5:** Summary Table of Recommendations from the Executive Courses on Genomics and Public Health Policy

**Recommendations to Harness Genomics for Health in Developing Countries**

**SCIENCE**
• Collaborate through national, regional, and international networks
• Survey and build capacity based on proven models through education, training, and needs assessments

**FINANCE**
• Develop regulatory and intellectual property frameworks for commercialization of biotechnology
• Enhance funding and affordability of biotechnology
• Improve the academic-industry interface and the role of small and medium enterprise

**ETHICS, SOCIETY, CULTURE**
• Develop public engagement strategies to inform and educate the public about developments in genomics and biotechnology
• Develop capacity to address ethical, social and cultural issues
• Improve accessibility and equity

**POLITICS**
• Strengthen understanding, leadership and support at the political level for biotechnology
• Develop policies outlining national biotechnology strategy

Already, the courses have spurred development of biotechnology capacity in the developing world. Beyond the generation of recommendations, the Courses produced a network for future collaboration. For example, an Indian participant invited to speak at the EMRO Course explained that although the Course occurred amidst Indian-Pakistani tensions, his presentation received a "warm response" from Pakistani delegates that led not only to the bulk transfer of the hepatitis B vaccines from India to Pakistan, but also the technology transfer that facilitated their manufacture in Pakistan [19]. "Every small cooperation matters," he said. "Science does not have borders." Beyond this example of collaboration, the EMRO Ministers of Health adopted the recommendations from that meeting and in the Latin America and Caribbean region, the Pan American Health Organization (PAHO) followed up with discussions of the recommendations from that event. The Courses have also stimulated international academic exchange – both bringing participants to Canadian institutions as well as funding graduate study abroad. Following the courses, both participants and the MRCGH staff have played advisory roles for one another in subsequent research projects.

We recognize that the structure of the Courses limits the rigor of the processes which generated these recommendations – although participants reached consensus through discussion, there was no formal consensus process. The Courses are rooted in opinions rather than economic or scientific analysis. Theoretically, had the Courses involved a different set of participants, the results might have differed. However, compared to the alternative of conducting surveys with 232 respondents from 58 countries, we feel the courses generated a more sustained engagement with participants.

This study serves to offer a taxonomy of potential actions for harnessing biotechnology to improve health; however, some countries are already attempting to deal with the challenges listed above. Ongoing studies at our Centre indicate that the Brazilian government has been trying to stimulate interactions between the public and private sector, specifically through the creation of an innovation law meant to facilitate interactions between academia and industry – due to this intervention, more and more private companies are tapping into services within universities for research and product development. With regard to the challenge of intellectual property management, a recent report from Médecins sans Frontières calls for developing countries to look to the success of Brazil and Thailand in issuing compulsory licenses [20].

These recommendations will be useful to all those interested in supporting science, technology, and innovation to improve health in the developing world – both for industrialized nations interested in supporting knowledge-based approaches to science and developing nations looking to foster biotechnology innovation. Across the sectors of academia, government, industry, and civil society, scientists, policymakers, regulators, venture capital firms, industry representatives, and donor communities will benefit from the applications of these insights.

Biotechnology can act both as a catalyst to foster overall development of science and technology as well as the development of practical solutions to local health needs. These recommendations align holistically and act as forces that will affect the development and adoption of health biotechnology in the developing world [21]. Applying these recommendations broadly across sectors and regions will empower developing countries themselves to harness the benefits of biotechnology and genomics for billions who have long been excluded.

## Competing interests

The author(s) declare that they have no competing interests.

## Authors' contributions

ASD and PAS designed and participated in all of the courses in addition to participating in the writing of this paper. DLP led in the Western Pacific and Southeast Asia Course and did the initial analysis of the recommendations. KB analyzed the recommendations and drafted the paper. All authors read and approved the final manuscript.

## Pre-publication history

The pre-publication history for this paper can be accessed here:


